# Drug susceptibility of a clinical isolate of *Balamuthia mandrillaris*, a pathogenic free-living amoeba

**DOI:** 10.1128/aac.01482-25

**Published:** 2025-12-10

**Authors:** Pratima Dubey, Porntida Kobpornchai, Nongnat Tongkrajang, Suwipa Chaiyaloom, Chenyang Lu, Christopher A. Rice, Kasem Kulkeaw

**Affiliations:** 1 Siriraj Integrative Center for Neglected Parasitic Diseases (SiCNPD), Department of Parasitology, Faculty of Medicine Siriraj Hospital, Mahidol University 65106, Bangkok, Thailand; 2 Siriraj-Long Read Lab, Faculty of Medicine Siriraj Hospital, Mahidol University 65106, Bangkok, Thailand; 3 College of Veterinary Medicine, Department of Comparative Pathobiology, Purdue University 70731, West Lafayette, Indiana, USA; 4 Purdue Institute for Drug Discovery (PIDD), Purdue University, West Lafayette, Indiana, USA; 5 Purdue Institute of Inflammation, Immunology and Infectious Disease (PI4D), Purdue University, West Lafayette, Indiana, USA; 6 Regenstrief Center for Healthcare Engineering (RCHE), Purdue University, West Lafayette, Indiana, USA; 7 Advanced Microfabrication and Biomaterials for Organ-on-Chip Research Unit (AMBiO), Mahidol University 26685, Salaya, Nakhon Pathom, Thailand; The Children's Hospital of Philadelphia, Philadelphia, Pennsylvania, USA

**Keywords:** *Balamuthia mandrillaris*, *Balamuthia *amoebic encephalitis, drug susceptibility, drug repurposing

## Abstract

*Balamuthia amoebic* encephalitis (BAE) is a highly fatal infection caused by *Balamuthia mandrillaris*, an amoeba that lives in soil and water. In Thailand, three fatal cases of BAE have been documented, but no survivors have been reported, raising questions about current treatment regimens. Previous drug repurposing studies reveal some potent pharmacological compounds, but the drug susceptibility of the clinical isolate of pathogenic amoeba remains variable. Given the success in isolating *B. mandrillaris* from the human biopsied brain, this study aims to assess the amoebicidal effect of several previously repurposed drugs and suggested therapies for BAE. The trophozoites of a new clinical isolate, the KM-20 strain, were exposed to 12 compounds, including pentamidine, the most widely used antiprotozoal drug, and nitroxoline, the recent radical cure for BAE. The amoebicidal effect was assessed using the ATP level as a cell survival biomarker. The circularity and surface area of the cells were used as recrudescence indicators. Among all drugs tested, nitroxoline is the most potent amoebicidal drug without recrudescence. Topical antiseptic agents caused amoeba lysis at all doses tested, suggesting potential use for cutaneous balamuthiasis. Compared with two laboratory-adapted V039 and PRA-291 strains, the KM-20 isolate had reduced drug susceptibility to all of the tested compounds, suggesting strain dependency of amoebicidal activity. This study provides drug susceptibility data against a novel and geographically diverse clinical isolate of *B. mandrillaris* to assist in prioritizing anti-*Balamuthia* agents for further drug development testing, followed by *in vivo* efficacy testing animal models before clinical trials and drug repurposing.

## INTRODUCTION

Several protist species inhabit soil and water, but some become a threat to human life if they enter the body. The most common pathogenic free-living amoebae identified as pathogens include *Naegleria fowleri*, *Acanthamoeba* spp., and *Balamuthia mandrillaris*. The two former genera are reported more frequently, while *B. mandrillaris* infection is currently rarer with a similar fatality. *B. mandrillaris* was first identified as an etiological agent in 1986 from the autopsied brain of a pregnant mandrill baboon who died of encephalitis (1). Humans are not the only accidental hosts. Other mammals, such as dogs, great apes, and tigers, have been reported to get infections (2). Clinical investigation leads to the hypothesis that *B. mandrillaris* enters the human body through a cutaneous lesion, skin cut, or nasal cavity, followed by invasion of the central nervous system, which can cause *Balamuthia* amoebic encephalitis (BAE). Pathological examinations of brains infected with *B. mandrillaris* reveal necrosis, hemorrhage, and macrophage–lymphocyte–predominant inflammation, termed granulomatous amoebic encephalitis (3). Young people, adults, and the elderly are at risk for BAE regardless of their immune status. Although BAE is rare, the environment poses a life-threatening threat, justifying the need for early and rapid diagnoses and better clinical therapies.

The clinical outcome of current BAE treatment remains uncertain and unpredictable. The US Centers for Disease Control and Prevention (CDC) recommend pentamidine isethionate, miltefosine, sulfadiazine, flucytosine, azithromycin, clarithromycin, and nitroxoline (4, 5). Various combinations of these drugs have been used clinically with BAE, and most of them have failed (6–8). Surviving cases always simultaneously underwent surgical removal of damaged brain tissue (9–13). Among the CDC-recommended drugs, only pentamidine isethionate, miltefosine, and nitroxoline were reported to have *in vitro* amoebicidal activity (14–18). Therefore, better therapies against *B. mandrillaris* infection are urgently needed.

Drug discovery and development is a long and expensive process. This includes multiple screening of various small-molecule libraries to identify hits, early- and late-stage hit-to-lead optimization, preclinical safety and efficacy animal studies, and human safety and efficacy studies (clinical trials). The recruitment of eligible participants for clinical trials of rare diseases is often challenging. To accelerate this process, clinically approved drugs with known pharmacokinetics and safety are subjected to *in vitro* drug susceptibility testing against *B. mandrillaris*. The two most common pathogenic strains of *B. mandrillaris* used in drug repurposing are those isolated from the brain tissue of a pregnant mandrill baboon (*Papio sphinx;* V039) and a 6-year-old girl (V451); both are grown in *B. mandrillaris* ITSON (BMI) complete media (19). Both pathogenic strains are now available in the American Type Culture Collection, designated as ATCC 50209 and ATCC PRA-291. Laurie et al. (17) screened more than 2,000 compounds of clinically approved drugs against the V451 strain and identified nitroxoline as a potent amoebicidal drug. Nitroxoline was repurposed as a combinational therapy, which has now been used successfully to the treatment of a BAE patient (20). Rice et al. (21) used high-throughput screening methods of the world’s largest available repurposed drug library—the Calibr library at Scripps ReFRAME—against the *B. mandrillaris* V039 strain and reported a set of potent lipid inhibitors, such as cerivastatin sodium, pitavastatin calcium, and bervastatin. Other studies identified specific drug targets from the phenotypic drug screening of amoebicidal compounds with known antiparasitic activity, including propamidine, a drug used to treat *Acanthamoeba* keratitis (22). Moreover, Colon et al. (23) screened libraries consisting of Food and Drug Administration–approved compounds and the Medicines for Malaria Venture Pathogen Box and identified the antifungal drug posaconazole as an amoebicidal drug against *N. fowleri* (23). They showed *in vivo* preclinical efficacy through a CD-1 ICR mouse clinical trial (MCT), with a combination of 20 mg/kg of posaconazole and 25 mg/kg of azithromycin curing ~65% of *N. fowleri* (ATCC 30215 – Nf69)–infected mice. Repurposing existing drugs also leads to the successful treatment of other infectious diseases, including anticancer drugs for HIV (24) and antimalarial drugs for COVID-19 (25). Therefore, drug repurposing/repositioning is a promising approach to identify potent drugs in a faster and cheaper period.

This study aims to test the susceptibility of a new clinical isolate of *B. mandrillaris*, the KM-20 strain, against clinically approved drugs. This *B. mandrillaris* strain was previously isolated from the biopsied brain of a fatal case of BAE in a 4-year-old Thai girl (26, 27). We selected pentamidine and miltefosine, the drugs commonly administered for standard BAE treatment; nitroxoline, the potent curative drug in a BAE patient; topical antiseptic compounds, which also exhibit amoebicidal activity; propamidine, an effective drug for *Acanthamoeba* keratitis; and statin and its derivative, which alter mevalonate/sterol synthesis. We did not include flucytosine, fluconazole, and sulfadiazine due to the low to no amoebicidal activity in previous *in vitro* studies (17, 22).

## MATERIALS AND METHODS

### Isolation of *B. mandrillaris* from the biopsied brain

A 4-year-old Thai girl was diagnosed with BAE based on histological examination and PCR. The biopsied brain tissue was subjected to isolation of the pathogenic amoeba (26, 27). Briefly, brain tissue was immersed in Dulbecco’s Modified Eagle Medium (DMEM) supplemented with 10% fetal bovine serum (FBS, Hyclone, Pasching, Austria), 500 units/mL penicillin, and 500 µg/mL streptomycin. The tissue was chopped using a sterile surgical blade and subjected to trypsin digestion. The sample was passed through sterile gauze pads to remove large debris. The cell suspension was centrifuged and washed three times with the above-mentioned media. The cell pellet was resuspended with the same medium and added to a well containing human lung carcinoma A549 cells. Through multiple passages with human A549 cells, slow-moving trophozoites were initially obtained. After continuous culture, the trophozoites became active in removing human cells and exhibited cytoplasmic protrusion. When the human cells were completely removed and the culture continued without medium change, the trophozoites changed morphology to a cyst stage. This laboratory-adapted *B. mandrillaris* has been named KM-20.

### Culture of *B. mandrillaris* trophozoites

Before the drug susceptibility test, the trophozoites of the *B. mandrillaris* KM-20 strain were cultured using BM-3 media without human cells (axenic culture). The trophozoites took a few months to proliferate in the axenic culture. The BM-3 medium consists of proteose peptone (4 g/L, TMMedia, Rajasthan, India), yeast extract (4 g/L, TMMedia, Rajasthan, India), torula yeast RNA (1 g/L, Sigma, City, Japan), ox liver digest (10 g/L, Himedia, Maharashtra, India), lipid mixture (1 g/L, Gibco, Grand Island, USA), hemin (2 mg/L, Sigma-Aldrich, Massachusetts, USA), taurine (50 µg/L, Sigma-Aldrich, Massachusetts, USA), and vitamin mixture (Gibco, Grand Island, USA) with Hanks’ balanced salt solution (Sigma-Aldrich, Saint Louis, The State) (28). Normally, logarithmic trophozoites were subcultured every 8 to 10 days when approaching ~80% confluence. For the typical cell culture and maintenance of *B. mandrillaris* on human cells, the trophozoites were cultured with a monolayer of human neuroblastoma SH-SY5Y or human lung carcinoma A549 cells. When the host cells were completely removed, the trophozoites were subcultured in a ratio of 1:5 or 1:10.

### Culture of human lung carcinoma and neuroblastoma cell lines

Human neuroblastoma SH-SY5Y cells (ATCC No. CRL-2266; ATCC, Manassas, VA) were maintained in a 1:1 ratio of the mixture of ATCC-formulated Eagle’s minimum essential medium and F12 medium supplemented with 10% FBS, 500 units/mL penicillin, and 500 µg/mL streptomycin. The cells were incubated in a humidified environment with 5% CO_2_ at 37°C. The medium was renewed every 4–7 days. The subculture was carried out when the cells achieved 70–80% confluence. In ratios between 1:20 and 1:50, the confluent cells were transferred to a new well as recommended by the ATCC protocol. The culture of the human lung carcinoma A549 cells used 10% FBS-supplemented Dulbecco’s Modified Eagle Medium. Cells were incubated at 37°C as mentioned above and subcultured when the cell density reached 60–80% confluence. Trypsin 0.05% was used to detach the cells from the well to harvest the adherent cells. Trypan blue was deployed to count viable cells using a hemocytometer under a light microscope. Human lung carcinoma A549 cells were subcultured at a 1:10 ratio per well of a 6-well plate.

### Growth curve of *B. mandrillaris* trophozoite

To examine the growth of the *B. mandrillaris* independent of the feeder cells, the trophozoites were cultured in the BM-3 medium under axenic condition. The trophozoites were initially seeded at the cell density of 10,000 cells/mL in a well of the 12-well plates, with a total volume of 1 mL per well. The *B. mandrillaris* trophozoites were continuously cultured for 19 days in different conditions: (1) the complete BM-3 medium without medium change; (2) the ox liver digest-depleted BM-3 medium; and (3) the BM-3 medium lacking yeast extract and glucose. All conditions were maintained without medium change but refilled with 0.2 mL of fresh medium every 7 days to prevent liquid evaporation. For sufficient nutrient control, the trophozoites were cultured with medium renewal on days 7 and 14. The number of parasites was counted in two-day intervals using a hemocytometer. The growth curve of the trophozoites was plotted using time on the x-axis, while the total number of trophozoites was on the y-axis. The doubling time of trophozoites was calculated using the formula: doubling time (DT) = T × (In (2)/In(Nt/N0)), where *T* is the time duration from initial seeding to the point at which fresh medium was added or changed—on day 7 for the initial condition and on day 11 onward for the post-condition change, *Nt* is the number of amoeba at time point (t), and *N0* is the number of amoeba at initial seeding.

### Drug susceptibility test

All compounds were dissolved in DMSO to a 10–35 mM stock. To determine the half minimum inhibitory concentration (IC_50_), each compound was diluted in BM-3 medium in a two-fold serial dilution. Final screening concentrations of 0.78–200 µM were tested for atorvastatin, atorvastatin calcium hydrate, simvastatin, chlorhexidine, chlorhexidine hydrochloride, chlorhexidine acetate hydrate, polyhexamethylene biguanide hydrochloride (PHMB HCl), miltefosine, pentamidine, pentamidine isethionate salt, and propamidine. In contrast, nitroxoline was diluted to 0.078–20 µM for screening. The selected doses of 0.78–200 μM fall within the same range as a previous study, in which 0.14–300 μM concentration of drugs were used (17). These drug dose ranges were not based on therapeutic concentrations. Instead, the highest dose is justified to achieve its cidal or inhibitory activity. In the negative control, the final concentrations of DMSO corresponded to match the final DMSO concentration for each diluted drug (usually 1% or lower). The *B. mandrillaris* trophozoites were seeded at 8,000 cells per 50 µL per well of a 96-well plate and incubated with 50 µL of each compound at 37°C for 72 h. The optimal seeding density was selected to achieve 70–80% confluence, which allowed morphological observation and cell proliferation during 72 h of drug exposure (Data S1). To measure intracellular ATP as a readout of cell survivability, 100 µL of CellTiter-Glo 3D Cell Viability reagent (Promega, Madison, Wisconsin) was added to each well and incubated at room temperature (25°C) for 25 min without shaking the plate. The end-point measurement of the luminescent signal (relative light unit, or RLU) was examined using a Biotek Synergy H1 multidetection microplate reader. Cell survivability was calculated by normalizing with the signal of the DMSO-exposed control. The percentage of survivability was calculated as: percent viability = [(test sample − blank control) / (negative control − blank control)] × 100. The media without trophozoites was used as a blank control. The IC_50_ curves were generated using the Levenberg-Marquardt algorithm and plotted with cell survivability versus drug doses. The IC_50_ values were calculated from three biological replicates and are reported as the mean ± standard error.

### Recrudescence assay

To confirm the amoebicidal effect of a given compound, recovery of drug-exposed trophozoites is used as an indicator. Coculture of the drug-exposed trophozoites with human non-cancerous cells is reportedly used to examine the recrudescence (17). In this study, the human cancer cell line was used for the recrudescence assay. However, the detachment of human cancer cells limits their use in long-term culture. Thus, the recrudescence of the drug-exposed amoeba was assessed on the basis of the recovery of the pleomorphic form and the cytoplasmic protrusion. A total of 8,000 trophozoites were prepared in 50 µL and seeded in a well of a 96-well plate. Then, the trophozoites were exposed to 50 µL of compounds: atorvastatin calcium hydrate (100, 50, and 25 µM), chlorhexidine (25, 12.5, and 6.25 µM), nitroxoline (20, 10, and 5 µM), pentamidine (100, 50, and 25 µM), and PHMB HCl (25, 12.5, and 6.25 µM) for 72 h. Following incubation, the remaining amoebae were washed using 1× PBS by centrifugation at 3,000 rpm at room temperature for 5 min and resuspended in a drug-free BM-3 medium. The compound-exposed amoeba was continuously cultured in a 6-well plate with BM-3 medium without the compounds until the day trophozoite recovery was observed. Cell morphology and movement were monitored daily. The transition from a round-shaped form to trophozoites was calculated by using circularity and surface area. To ensure the loss of recrudescence, the compound-exposed amoebae from the endpoint of the experiment were collected from the axenic culture in BM-3 medium and washed once with PBS. After resuspending the pellet with the culture medium of the host cells, the amoebae were plated onto the monolayer of A549 cells and continuously cultured for 3 days. The morphology of the amoeba and the clearance of the host cells were observed daily. The recrudescence assay was performed with three independent biological replicates.

### Cerebral organoid and drug test

To test the effect of a drug on reducing brain damage from amoeba, a cerebral organoid model was used for these tests. Human iPSC culture and brain organoid generation were performed according to the previous report (29). Briefly, a stepwise protocol was performed to obtain differentiated cell stages, including embryoids, neuroepithelial cells, neural stem cells, and mature neurons (30). The protocol follows the manufacturer’s instructions for the STEMdiff Cerebral Organoid Kit (STEMCELL, Canada). Before the drug test, cerebral organoids were cocultured with *B. mandrillaris* trophozoites KM-20 at 37°C and 5% CO_2_ for 3 days to allow cell damage. Drug exposure with 100 µM miltefosine, 35 µM nitroxoline, or vehicle control (2% DMSO) was added to the coculture for 20 h (17, 29). After drug removal, the organoids were continuously cultured for 16 days with a renewal of the medium every 4 days. Three biological replicates were performed. Morphological changes in cerebral organoids were observed daily under an inverted microscope, and the surface area was calculated using ImageJ.

### ELISA

Cell damage to the human cerebral organoid was evaluated using two specific brain trauma biomarkers: ubiquitin carboxyl-terminal hydrolase family 1 (UCH-L1) and glial fibrillary acidic protein (GFAP) as an indicator of neuronal cell body damage and astroglial injury, respectively (31–33). Briefly, the culture medium was collected every four-day interval until 16 days after the coculture with trophozoites and drug exposure. The ELISA was performed according to the manufacturer’s instructions. The ELISA kit for UCH-L1 was from Thermo Fisher Scientific (Frederick, MA), and the kit for GFAP was from Abcam (Cambridge, UK).

### Statistical analysis

All graphs were created using GraphPad Prism 8 software (GraphPad Software Inc., La Jolla, CA, USA). Statistical analyses were performed in SPSS version 18 (IBM Corp., Armonk, NY, USA). The data normality was evaluated with the Kolmogorov–Smirnov test. Differences among groups were evaluated by one-way ANOVA followed by Bonferroni’s multiple-comparison test. The significance of the differences between the groups was recorded as a *P-*value = <0.05 (*), <0.001 (**), and <0.0001 (***).

## RESULTS

### Morphology and proliferation of *B. mandrillaris* trophozoites in cell culture

**Fig 1:**
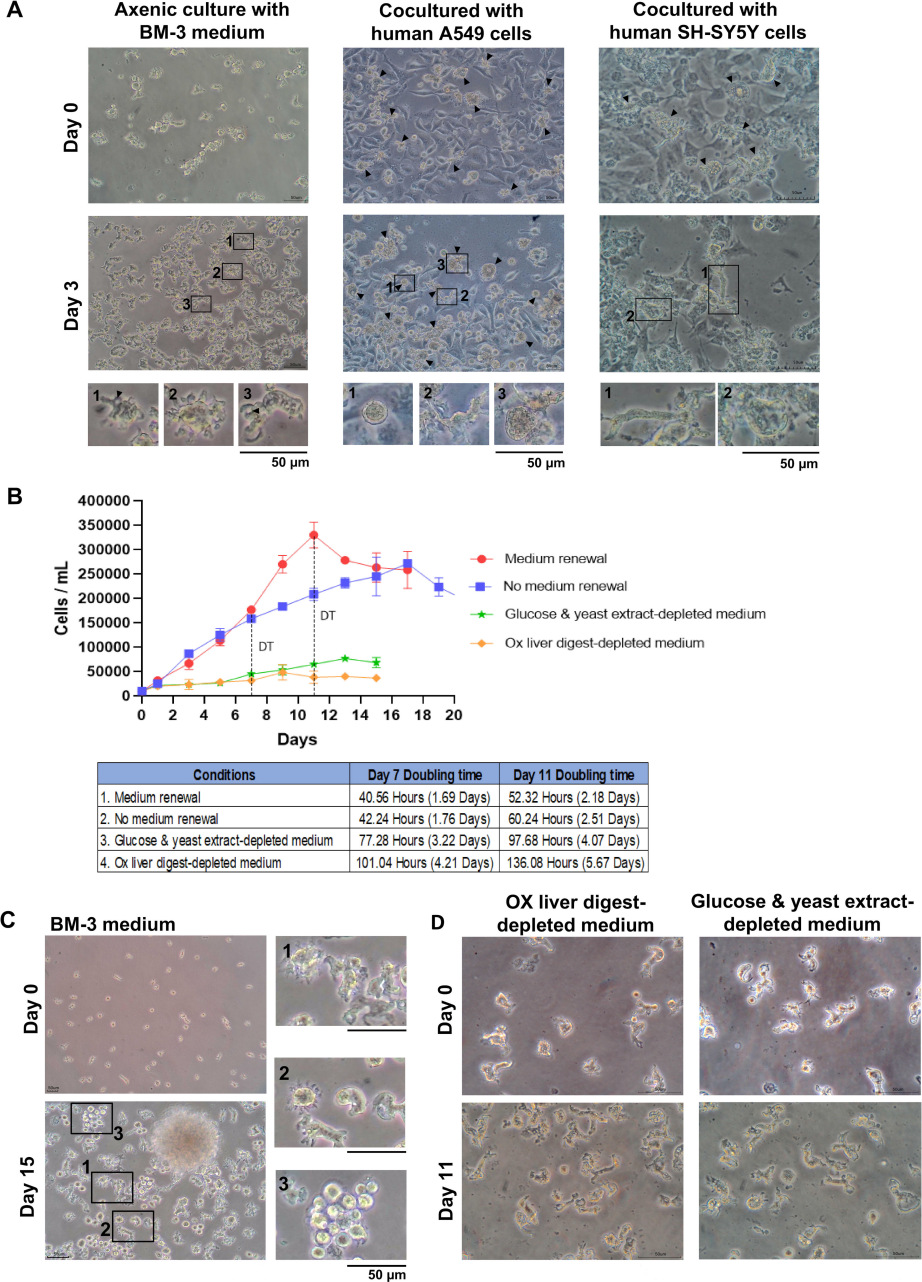
Growth and proliferation of the clinical isolate of *B. mandrillaris*. Trophozoites were cultured in a well of the 6-well plate at a cell density of 10,000 cells/mL. During continuous culture, viable trophozoites were counted using trypan blue and a hemocytometer. (**A**) Microscopic observation of *B. mandrillaris* under three different culture conditions. In the left subpanel, the trophozoites were cultured without feeder cells, known as “axenic culture,” using BM-3 medium. Representative images of trophozoites are shown at higher magnification (inlets 1–3). Arrowheads in these inlets point to vacuoles. Middle subpanel, the coculture of the *B. mandrillaris* with human lung carcinoma A549 cells as feeders. Arrowheads indicate the amoeba. The inlets 1, 2, and 3 show a cyst, elongated form, and round shape of the *B. mandrillaris*, respectively. Right subpanel, the coculture of the *B. mandrillaris* with human neuroblastoma SH-SY5Y cells. Arrowheads indicate the trophozoites, while two distinct forms were noted at inlets 1 and 2. Scale bars = 50 µm. (**B**) Growth curve of *B. mandrillaris* trophozoites in feeder-free BM-3 medium. The trophozoites were continuously cultured without medium change for 19 days (blue line). Cells were counted every two-interval days starting from day 1 after culture. The BM-3 medium was renewed on days 7 and 14 (red line). The effect of ox liver digest, yeast extract, and glucose depletion on cell proliferation was examined (orange and green lines, respectively). Three technical replications were performed. Doubling time (DT) was calculated from the initial time point to day 7 and day 11. (**C**) Morphological changes of trophozoites in a continuous culture with BM-3 medium. Without medium renewal, the trophozoites remained active in projecting the short, spike-like pseudopods (inlets 1 and 2). Some become round without double layer walls and tend to clump (inlet 3). Scale bars = 50 µm. (**D**) Depletion of ox liver digest and yeast extract from BM-3 medium. Removal of either ox liver digest or yeast extract impaired proliferation but did not induce encystment (left and right subpanels, respectively). The trophozoites become thin, elongated shapes with a smaller number of pseudopods. Scale bars = 50 µm.

After several months of continuous culture, the *B. mandrillaris* KM-20 strain adapted well to survive and proliferate under standard cell culture conditions. In a host cell-free (axenic) culture using BM-3 medium, the *B. mandrillaris* trophozoites display irregular pleomorphic shapes with size variations (left subpanels, Fig. 1A). Size measurement was performed by selecting microscopic areas, where the trophozoites were confluent and the edges of single cells could be clearly defined (Data S2A). The length of the trophozoites ranged from 11 to 40 µm with a mean of 23.12 µm and a mode of 11.38 µm. Most cells exhibited prominent pseudopodia surrounding the cells (the magnified inlets 1-3 in Fig. 1A). The cytoplasm is rough and granular with vacuoles (arrows in the magnified inlets 1 and 3, Fig. 1A). *B. mandrillaris* trophozoites remain proliferative even without medium change up to 19 days (blue line, Fig. 1B). The doubling time (DT) of the trophozoite was 42.24 h (~1.76 days). Interestingly, when the BM-3 medium was changed on day 7, the trophozoites increased more than the trophozoites cultured without medium change, but only for an additional 40.56 h (~1.69 days). On day 11, the doubling time of these trophozoites was 52.32 h (red line, Fig. 1B). Following 15 days of cultivation without medium renewal, the trophozoites aggregated as cell clumps (Fig. 1C). In the cell clump, each trophozoite became round with a less elongated pseudopod (the magnified inlet 1-2, Fig. 1C). Instead, it had a protruding, spike-like cytoplasm (the magnified inlet 2, Fig. 1C). However, the incomplete cyst-like structure of *B. mandrillaris* was observed as a round shape without a double-layered cell wall when the culture was continued for 15 days without a medium change and the cell density exceeded 2.5 × 10^5^ cells/mL (the magnified inlet 3, Fig. 1C). As expected, the removal of ox liver digest, glucose, and yeast extract severely impaired trophozoite proliferation (orange and green lines, respectively, Fig. 1B). However, the trophozoites never transformed into cysts, even with the removal of key components from the BM-3 medium (Fig. 1D). In contrast to axenic culture in BM-3 medium, the morphology of the trophozoite differed when cocultured with human lung A549 carcinoma cells and SH-SY5Y neuroblastoma cells. The morphology of the trophozoites was indistinguishable between either mammalian cell type (Fig. 1A). On day 3 of the coculture, an area without human cells was observed (Fig. 1A). A cyst with a double-layer wall could be observed (the magnified inlet 1 of the middle subpanel, Fig. 1A). There were two main forms of the trophozoites: (1) elongated with long pseudopodia (the magnified inlet 2 in the middle subpanel and inlet 1 in the right subpanel) and (2) round without cytoplasm protrusion (the magnified inlet 3 in the middle subpanel and the magnified inlet 2 in the right subpanel). In general, the variable morphologies of trophozoites depend on their culture condition. The transformation into the cyst-like structure occurs in unfavorable culture media, suggesting nutritional insufficiency with 10% DMEM with A549 cells.

### Amoebicidal activity of pharmacological agents

**Fig 2:**
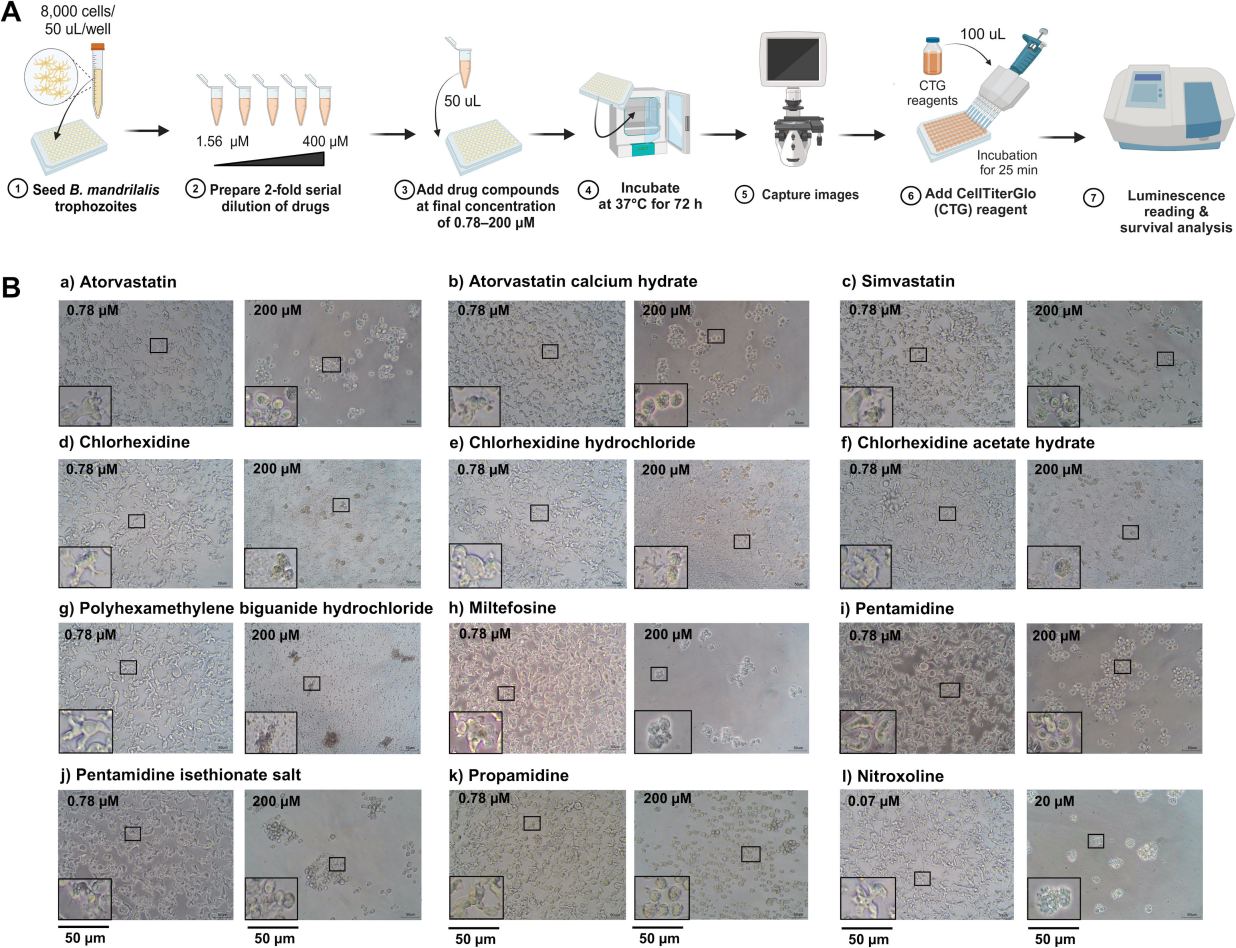
Drug susceptibility assay. (**A**) Schematic diagram illustrating steps in the drug test. The trophozoites were plated into a 96-well plate, followed by the addition of an equal volume of drug to obtain final concentrations of 0.78-200 µM. After 72 h of incubation, the total level of ATP was measured using CellTiter-Glo (CTG) reagent. The luminescence signal was subjected to calculate half inhibitory concentration (IC_50_). The luminescence signal from the vehicle control experiment was used to calculate cell survival. (**B**) Representative images of trophozoites exposed to 0.78 and 200 µM of each drug. The inlets in each image show the cell morphology at higher magnification. Three technical replicates were performed. Scale bars = 50 µm.

To test and compare amoebicidal activity, the trophozoites of the *B. mandrillaris* KM-20 strain and “reference” strains, including V039 and PRA-291, were exposed to 12 pharmacological agents (Table 1). Final concentrations, ranging from 0.07 µM to 20 µM or 0.78 µM to 200 µM, were incubated with the trophozoites for 72 h (Fig. 2A). Compounds were categorized into different action modes, including sterol synthesis inhibitors, topical antiseptic activity, anti-intracellular parasites, antifungal compounds, and antibacterial activity, respectively (Table 1). The half-maximal inhibitory concentration (IC_50_) values were measured by plotting the dose-response curve between percent cell viability and drug concentration (Data S3). The most potent amoebicidal compound against *B. mandrillaris* trophozoites KM-20 was nitroxoline, with an IC50 of 9.21 ± 2.53 µM, with the IC_50_ values for other suggested therapeutics being higher, including miltefosine (128.4 ± 7.01 µM), pentamidine (50.14 ± 5.28 µM), PHMB HCl (14.57 ± 11.03 µM), and propamidine (20.15 ± 1.55 µM). Furthermore, the statin derivatives exhibited poorer amoebicidal effects with IC_50_ values of atorvastatin, atorvastatin calcium hydrate, and simvastatin being six times higher than that of nitroxoline (Table 1). Notably, the IC₅₀ values of all drugs against the *B. mandrillaris* V039 and PRA-291 strains were always lower than those for KM-20. To examine the phenotypic response of the trophozoites to each compound, we also observed the morphological changes under a light microscope. The vehicle-treated amoebas were used as a negative control, which did not alter the trophozoites’ morphology compared to BM-3 culture (Data S4). The trophozoites exposed to high doses of sterol synthesis inhibitors (a, b, and c in Fig. 2B), pentamidine (h in Fig. 2B), pentamidine isethionate salt (i in Fig. 2B), and propamidine (l in Fig. 2B) become round shapes without pseudopods. In contrast, cell debris was observed after trophozoites were exposed to topical antiseptic compounds: chlorhexidine, chlorhexidine hydrochloride, and chlorhexidine acetate hydrate (d, e, and f in Fig. 2B) and PHMB HCl (k in Fig. 2B). Miltefosine and nitroxoline exposure resulted in the deformation of the trophozoites without cell debris (g and j in Fig. 2B). However, these results indicate that most of the compounds induced incomplete cyst-like structures based on the lack of a double layer of cyst walls with a round shape.

**TABLE 1:** Half-maximal inhibitory concentration (IC_50_) values of all tested compounds against *Balamuthia mandrillaris*

Categories of drugs	Name of compounds	Therapeutic used	IC_50_ ± SD (µM)		
			CDC: V039	ATCC: PRA-291	KM.20
Cholesterol synthesis inhibitors	Atorvastatin	Prevent heart disease	1.1 ± 0.08	6.15 ± 0.49	97.22 ± 5.31
	Atorvastatin calcium hydrate	Reduce cholesterol, apolipoprotein, and triglycerides	1.12 ± 0.09	6.22 ± 0.79	60.69 ± 9.56
	Simvastatin	Treat cholesterol and triglyceride	2.48 ± 0.29	6.07 ± 0.31	54.25 ± 2.36
Antiseptic (topical)	Chlorhexidine	Treat gingivitis	0.92 ± 0.1	0.39 ± 0.06	16.73 ± 1.02
	Chlorhexidine hydrochloride	Redness of gums	0.85 ± 0.11	0.32 ± 0.07	16.27 ± 1.66
	Chlorhexidine acetate hydrate	Clean skin injury	0.94 ± 0.1	0.35 ± 0.08	23.88 ± 2.78
	Polyhexamethylene biguanide hydrochloride (PHMB HCl)	Treat wound infection and *Acanthamoeba* keratitis	12.8 ± 0.55	12.23 ± 0.72	14.57 ± 11.03
Anti-intracellular parasites	Miltefosine	Treat leishmaniasis	38.2 ± 3.72	26.93 ± 3.23	128.4 ± 7.01
	Pentamidine	Treatment of pneumocystis pneumonia, leishmaniasis, and trypanosomiasis	3.5 ± 0.09	1.04 ± 0.15	50.14 ± 5.28
Antifungal	Pentamidine isethionate salt	Prevent pneumocystis pneumonia	4.21 ± 0.55	1.32 ± 0.19	52.63 ± 7.81
Antibacterial	Propamidine	Treat *Acanthamoeba* keratitis	5.15 ± 0.48	3.01 ± 0.48	20.15 ± 1.55
	Nitroxoline	Urinary tract infection	3.34 ± 0.32	2.59 ± 0.34	9.21 ± 2.53

### Recrudescence of *B. mandrillaris* trophozoites after drug exposure

**Fig 3:**
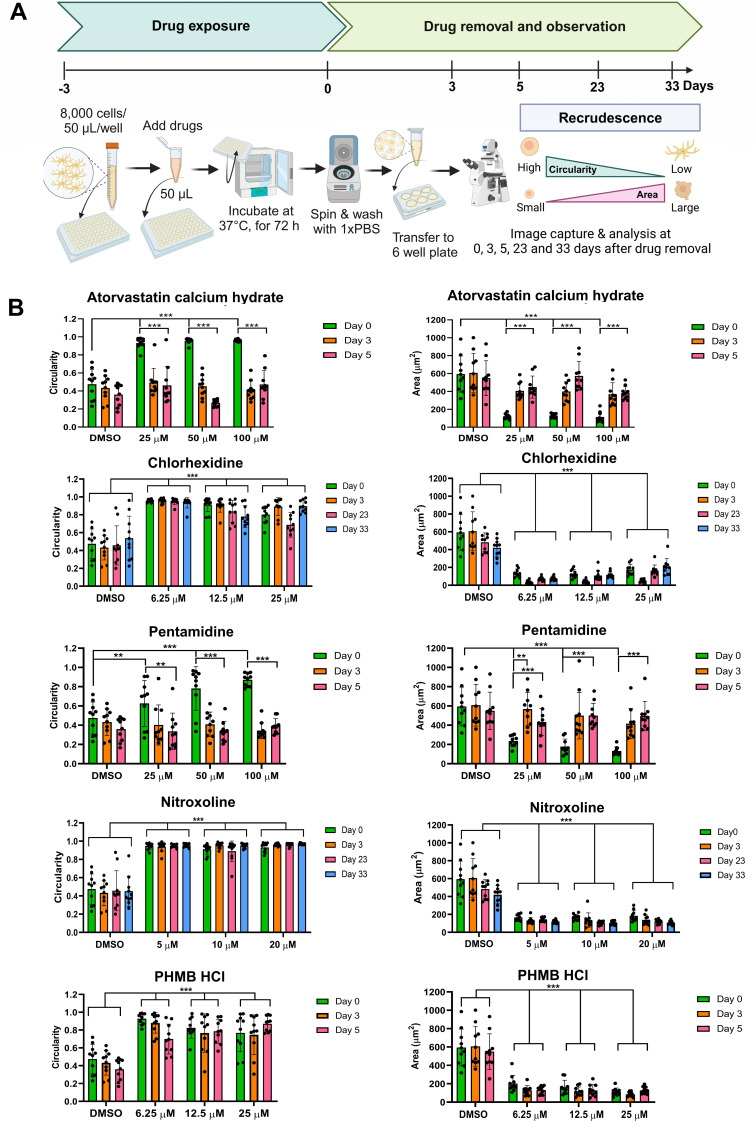
Amoeba recrudescence assay. (**A**) Schematic diagram illustrating the steps in the drug test. *B. mandrillaris* trophozoites were first exposed to three doses: 0.4–0.5×, 0.8–1×, and ~ 2× IC_50_ of the drugs for 72 h (*t* = −3 to 0 days). The trophozoites were then washed to remove the drugs (*t* = 0 days), cultured without drugs, and observed under the microscope (*t* = 0 to 33 days). To define a recrudescing cell, the morphological changes from a round shape to an elongated form with cytoplasm protrusion were examined. A decrease in cell circularity and an increase in the surface area indicate the recrudescing cells. (**B**) Bar graph of cell circularity and surface area. Each dot represents an individual trophozoite in an experiment. In the vehicle control experiment (0.2 and 2% of DMSO exposure), the cell circularity and surface area of the trophozoites remained constant until 33 of the recrudescence culture (blue bars in the chlorhexidine and nitroxoline-exposure experiments). Atorvastatin-, pentamidine-, and polyhexamethylene biguanide hydrochloride (PHMB HCl)-exposed trophozoites could recover within 3 days of the assay (orange bars). In contrast, the trophozoites exposed to chlorhexidine and nitroxoline remained unchanged on days 23 and 33 (pink and blue bars). The statistical analysis was performed by a one-way ANOVA test with Bonferroni’s multiple comparison. The significance of the data is shown as **P < 0.01 and ***P < 0.001, respectively.

The detachment of human cancer cells limits their use in long-term culture. Thus, the recrudescence of the drug-exposed amoeba was assessed based on the recovery of the pleomorphic form and the cytoplasmic protrusion. Some drugs caused most trophozoites to exhibit a round shape without a cyst wall-like structure. Therefore, we reasoned that a decrease in cell circularity and an increase in surface area, together with a pseudopod, can suggest recovery (Fig. 3A). A representative drug from each group was selected to test recrudescence, including atorvastatin, chlorhexidine, pentamidine, nitroxoline, and PHMB HCl. *B. mandrillaris* trophozoites were first exposed to three doses: ~0.4–0.5×, 0.8–1×, and ~2× IC_50_ of the drugs. The drugs were then removed, and the trophozoites were cultured without drugs for 3, 5, 23, and 33 days. The trophozoites exposed to pentamidine quickly returned to their pseudopod-projecting pleomorphic form within 3 days after drug removal (Data S5). When counting individual trophozoites, cell circularity decreased significantly, while surface area increased on days 3 and 5 post-culture under drug-free conditions (Fig. 3B). For sterol synthesis inhibitors, such as atorvastatin calcium hydrate, the round-shaped trophozoites transform to an elongated form on day 3 of the recrudescence assay (Fig. 3B). Cell debris was obtained after topical antiseptic compound exposure (chlorhexidine and PHMB HCl). We did not observe the intact form of the trophozoites during the 33 days of chlorhexidine and 5 days of PHMB HCl with drug-free BM-3 culture. Similarly, other pentamidine- or nitroxoline-exposed trophozoites become round without a pseudopod. In contrast, with the removal of nitroxoline, the rounded and shrunken form of amoeba remained unchanged up to day 61 of the drug-free BM-3 culture, indicating no recrudescence (Data S6A). Nevertheless, re-coculture with human A549 cells failed to show recrudescence of the nitroxoline-exposed *B. mandrillaris* trophozoites (Data S6B). In DMSO, there are no significant changes in cell circularity from days 0 to 33. Although some of the trophozoites transformed into cyst-like structures during long-term culture from days 11 to 33, the ratio between round and elongated shapes is low (Data S7). Thus, nitroxoline, chlorhexidine, and PHMB HCl pose amoebicidal and recrudescence inhibitory effects.

### Effect of the drug on the integrity of the cerebral organoid

**Fig 4:**
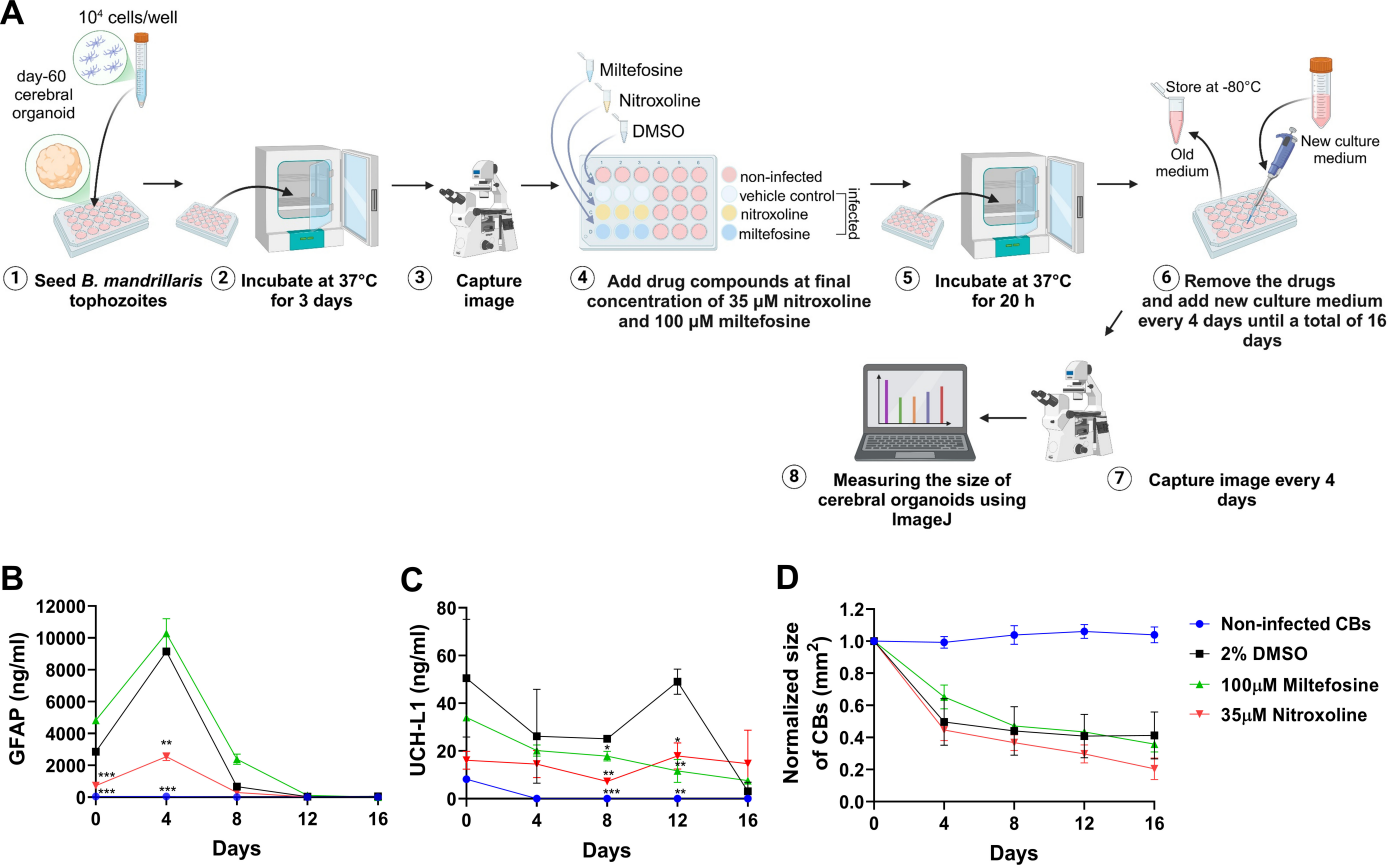
Effect of the drug on the cytotoxicity of *B. mandrillaris* trophozoites. (**A**) Schematic diagram illustrating the drug test in coculture with human cerebral organoids (CBs). The human 60-day CBs were cocultured with trophozoites for 3 days followed by drug exposure for 20 h. After drug removal, the CBs were cultured for 16 days with renewal media every 4 days. The culture media was collected to evaluate brain trauma biomarkers. The surface area of the human CB was calculated using ImageJ and displayed as mm^2^. Three biological replicates were performed. (**B and C**) The level of human GFAP and UCH-L1 in the culture media of the infected organoid postexposure with nitroxoline and miltefosine at 0, 4, 8, 12, and 16 days. (**D**) Changes in the surface area of the CBs. Data are mean ± SD. Three biological replicates of CBs were examined. Statistical analysis was performed using a one-way ANOVA test with Bonferroni’s multiple comparison. The significance of the data is shown as *P < 0.05, **P < 0.01, and ***P < 0.001, respectively.

In a previous drug repurposing study, Laurie et al. (17) used donor-derived brain explant tissue to demonstrate the amoebicidal activity of nitroxoline. Ameliorating fragmentation of brain explant tissue allows the assessment of the therapeutic effect of nitroxoline. We previously demonstrated that stem cell-derived cerebral organoids (CBs) could also be used to test nitroxoline as an alternative to brain tissue. The levels of brain trauma biomarkers GFAP and UCH-L1 were elevated following coculture with *B. mandrillaris* KM-20 trophozoites. With a minor modification of the protocol, day-60 cerebral organoids were first cocultured with trophozoites for 3 days, followed by exposure to drugs: 100 µM miltefosine or 35 µM nitroxoline for 20 h (Fig. 4A). After drug removal, the culture medium was collected, and the sizes of the organoids were measured every 4 days until 16 days. Without the amoeba, there were no detectable levels of GFAP and UCH-L1 in the culture media (blue line, Fig. 4B and C, respectively). After coculture of the CBs with trophozoites, the levels of GFAP and UCH-L1 increased significantly in the media (black line, Fig. 4B and C) compared to the non-infected CBs (blue line, Fig. 4B and C). Following 20 h of drug exposure, miltefosine could not reduce GFAP levels (day 0 in the green line, Fig. 4B) compared to the infected CBs exposed to the vehicle control (black line, Fig. 4B). Immediately after 20 h of drug exposure, the level of GFAP was significantly lower in the nitroxoline-treated CBs (day 0 in the red line, Fig. 4B) than in the vehicle and miltefosine treatment. Nitroxoline-mediated reduction in GFAP continued until day 4 after treatment. A significant decrease of UCH-L1 was observed on days 8 and 12 after exposure to miltefosine and nitroxoline (green and red line, Fig. 4C). The organoids were smaller in size and became translucent in coculture with the trophozoites (Fig. 4D; Data S8). By following the morphological changes at a four-day interval (days 4 to 16), we observed no significant difference in the size of the *B. mandrillaris*-cocultured cerebral organoids between the drug-treated and vehicle control groups (Fig. 4D; Data S8). Furthermore, the cytotoxicity of the drug-only group on the organoids was confirmed by more than 100% cell viability (Data S9). Therefore, miltefosine and nitroxoline could ameliorate tissue damage caused by the three-day preculture with *B. mandrillaris* trophozoites.

## DISCUSSION

Currently, there are only a few studies reporting the drug susceptibility of *B. mandrillaris* isolated from clinical BAE samples. This led to the discovery of nitroxoline as a potent radical cure for BAE. Consistent with Laurie et al., nitroxoline has an amoebicidal effect against the *B. mandrillaris* KM-20 strain that is isolated from human brain tissue. We could observe the rounded and shrunken forms following nitroxoline exposure. Moreover, none of the nitroxoline-exposed amoebas could recover after drug removal, which implies amoebicidal and no recrudescence activity. All topical antiseptic drugs cause *B. mandrillaris* cell lysis, suggesting a possibility of use for cutaneous balamuthiasis. However, the treatment with nitroxoline reduced the cytopathology of *B. mandrillaris* in our cerebral organoid culture.

Despite a nonstandardized protocol, most *in vitro* drug or chemical compound tests rely on the use of IC_50_ as an indicator of amoebicidal activity. Considering the most common drugs in BAE treatment, the IC_50_ values of nitroxoline, pentamidine, and miltefosine vary greatly. Laurie et al. (17) used a clinical isolate from the human brain and obtained an IC_50_ of 2.84 µM for nitroxoline. In comparison, the IC_50_ of nitroxoline against *B. mandrillaris* KM-20 strain is 9.21 ± 2.53 µM, which is 3.23× higher than the response of V451 and 2.76× and 3.55× higher than the responses of V039 and PRA-291 in this study, respectively. In contrast, Ahmad et al. (16) used the strain isolated from the autopsied brain of a pregnant mandrill baboon, reporting IC₅₀ values of 250 µM for miltefosine and 500 µM for pentamidine isethionate (16). This difference in drug susceptibility is also observed for miltefosine, with IC_50_ values ranging between 62.98 and 9.15 µM for miltefosine and pentamidine isethionate (17). Different protocols and drug susceptibility measurements are reported, resulting in potential confounders, such as cell number, volume, growth phase, and cell survival indicators. In addition, the culture conditions in BM-3 medium with depletion of certain nutrients, such as ox liver digest, glucose, and yeast extract, can reduce the growth rate. For this reason, the anti-amoebic response of most compounds tested under different medium conditions, such as *B. mandrillaris* ITSON (BMI) medium, presented a potent efficacy against *B. mandrillaris* (21). However, these factors can be controlled in *in vitro* drug testing. The most often used method is to set up 100% survival in the vehicle control experiment, in which all variables are controlled, except doses and types of drugs. Similar to *Acanthamoeba* spp., the drug susceptibility of *B. mandrillaris* varies, depending on the strains used and the *in vitro* assay (14, 15). Thus, variation in drug susceptibility is possibly due to the strain used, genotypic differences, and culture conditions. Consequently, the use of the standard protocol under the same conditions and the assessment methodology should be emphasized to determine the true efficacy and data for a better understanding of susceptibility profiles.

Currently, very few laboratory-adapted strains are available to the community. This is because misdiagnosis and isolation from clinical samples (human or animal cases) is a very time-consuming procedure, requires highly skilled staff, and has low success rates of axenic culture adaptation for these organisms. The first pathogenic strain of *B. mandrillaris* was isolated from the autopsied brain of a pregnant mandrill baboon diagnosed with GAE. Now, it was established as the CDC: V039 strain and deposited in ATCC with code 50209 (1, 34). There are other *B. mandrillaris* strains deposited in the ATCC, including the V451 strain (code PRA-291) from New York, United States, and the V416 strain (code PRA-290) from Queensland, Australia. Although more than 100 BAE cases were reported, not all cases are subjected to isolation and *in vitro* culture adaptation. Our success in isolating pathogenic strains is probably not due to the burden of amoeba in the brain, since only a single trophozoite was observed in the biopsied brain section. Immediate single-cell preparation of unfrozen tissue and coculture with human cells probably contributed to our success. It took 7 to 8 months of continuous culture with human lung carcinoma A549 cells until the trophozoites could proliferate and destroy the human cell in a few days to a week. In the presence of human feeder cells, the trophozoites of the *B. mandrillaris* KM-20 strain exhibit morphological differences compared to the axenic culture. Both lung carcinoma A549 and neuroblastoma SH-SY5Y cells adhere and grow as monolayers. Since the medium supplemented with fetal bovine serum provided fewer nutrients than BM-3, human feeder cells likely serve as an alternative nutrient source. This additional may promote cytoplasmic extension and adhesion of the trophozoites (26).

Historically, the CDC: V039 strain was isolated from minced brain tissue that was cocultured with monkey kidney E6 cells but not the human MRC-5 cell line (1). It took 3 weeks for the amoeba to first appear under a microscope on the monolayer of E6 cells. Visvesvara et al. (1) continued to culture for six to eight consecutive months until the amebae were able to destroy the monolayered E6 cells in 5 to 7 days. This strain is 15–60 µm in length with an irregular shape. Similarly, the size of the KM-20 strain varies from 11 to 40 µm. After 12% galactose exposure, the V039 strain forms cysts in the axenic culture. The cysts have a single nucleus and are round, with a diameter of 15–30 µm and two walls. Similarly, *B. mandrillaris* cysts are 9–20 µm in diameter. The morphology of the trophozoite and cyst is not indistinguishable between the CDC: V039 and KM-20; however, mitochondrial genome-based phylogenetic analysis suggests genetic differences (27). In the previous study, when the *B. mandrillaris* trophozoites are exposed to various drugs, they undergo encystment. *B. mandrillaris* cysts could resume their growth as trophozoites after drug removal and coculture with human feeder cells (17). Hence, the cysts represent the resistant form against the particular drug. In this study, the round-shaped structures were observed under a microscope, but without a double-layered cell wall. After drug removal and axenic culture, the cyst-like form becomes an active trophozoite, implying the incomplete encystment. Consistent with previous and recent studies, this cyst-like morphology is potentially similar to *Acanthamoeba*’s pseudocyst (35, 36). Moreover, environmental isolates of *B. mandrillaris* have been reported to harbor endosymbiotic bacteria (37). There is growing evidence that the environmental and clinical isolates of the keratitis-causing *Acanthamoeba* spp. also harbor endosymbiotic bacteria (38, 39). Using the same primer sequences specific to bacteria 16s rRNA, the PCR and DNA sequencing showed amplification of the 16s rRNA gene, which is located in the mitochondrial genome of the *B. mandrillaris* KM-20 strain (Data S10). This indicates that there are no bacterial endosymbionts in the *B. mandrillaris* KM-20 isolate. It should be noted that the KM-20 strain was obtained from a clinical brain sample and has been cultured *in vitro* for a few years. Thus, the lack of endosymbiotic bacteria in the *B. mandrillaris* KM-20 strain is possibly due to origin differences and the *in vitro* selection pressure.

Drug susceptibility indicates whether a pathogen can survive in the presence of a drug at specific concentrations. Survivability is generally assessed based on the number of amoebae remaining after drug exposure. Generally, surviving microorganisms are active in metabolism, continuously producing ATP. Therefore, intracellular ATP has been widely used as a readout of cell survivability (40). Nevertheless, the metabolism-inactive dormancy stage involves a decrease in ATP production, which can be confounded by the transition to dormancy or a dormancy-like stage. Therefore, the recovery of the drug-exposed amoeba or recrudescence is examined and allows a distinction between the amoebicidal and the amoebistatic effects, in which the amoeba remains viable but is not metabolically active. The recrudescence assay is reported to be performed by counting the period during which the drug-exposed amoeba removes all mammalian cells in a given well. The period varies depending on the extent to which the drug alters the cellular functions of the amoeba. Human cancer cell lines are used as nutrient sources and grow as monolayers. The overgrowth of cancer cells results in self-detachment from the well. Moreover, trophozoites that are exposed to a drug are maintained in host cell-free condition. It will take longer for the trophozoites to adapt to a coculture with human cells, in which the culture medium differs. Thus, the complete removal of monolayered human cancer cells can be confounded by overgrowth and changes in the microenvironment surrounding the amoeba. In this study, we reasoned that recovery of the trophozoites can be conducted in the feeder-free medium and monitored by observing morphological changes and measuring the circularity and area of amoebae. We did not conduct the recrudescence assay through co-culturing with mammalian cells because the host cells detached, whereas the amoebas remained in a cyst-like structure. In the drug susceptibility assay, some disinfectants cause trophozoite lysis, leaving cell debris in wells. These “drugs” included chlorhexidine, chlorhexidine acetate hydrate, chlorhexidine hydrochloride, and PHMB HCl. Among some drugs, the trophozoites become round but without a double-layered cell membrane (incomplete cyst-like structure). After drug removal, round-shaped trophozoites start to protrude pseudopods and change to an irregular shape with branches of cytoplasm protrusion, reflecting their typical morphology. This morphological change decreases roundness while increasing surface area. Thus, we propose using the decrease in cell roundness and increase in surface area as indicators of viable amoeba recovery. This method does not require the coculture with human cells and takes a shorter period for cellular assessment. However, the recrudescence may be affected by treatment at lower drug concentrations. Interestingly, even low concentrations of nitroxoline under the IC_50_ could delay the recrudescence, investigating the incomplete cyst-like structure of amoeba up to 61 days, without eliminating host tissue. This finding is supported by a study of the effect on host tissue damage in the nitroxoline-treated cerebral organoids (29). Nitroxoline prevented traumatic brain injury by reducing the protein levels of biomarkers for neuronal cell body injury (UCH-L1) and astroglia injury (GFAP).

Despite utility in assessing brain damage inhibition of anti-amoebicidal drugs, the use of cerebral organoids faces limitations, particularly the lack of blood-brain barriers and immune cells (29, 41). In the first cell differentiation process, human pluripotent stem cells first formed the embryoid bodies consisting of the ectoderm, mesoderm, and endoderm. The subsequent phases selectively induced ectoderm to differentiate into the neuronal cell type. The protocol deployed in this study did not include the induction of mesoderm to form vasculature nor incorporate immune cells (42). Thus, the human cerebral organoid is more applicable for testing neuronal cell damage rather than assessing damage on blood-brain barrier or studying immune responses.

Regarding the clinical translation, the IC_50_ values of all drugs against *B. mandrillaris* strain KM-20 are two times higher than those of CDC: V039 and PRA-291 “reference” strains. Thus, the strain dependency of the amoebicidal drug doses could explain the variability in therapeutic outcome among the BAE cases and patient outcomes worldwide. In drug discovery, getting therapeutics into the brain is one of the most difficult challenges, especially at concentrations that have log efficacy against pathogenic free-living amoeba. Through a review of the literature, we found studies documenting cerebrospinal fluid or maximum plasma concentration (plasma Cₘₐₓ) values for compounds with drug-like activity <10 µM against one or more *Balamuthia* strains. From the reviewed results, we can conclude that single doses of various compounds result in a range of 4.5- to 1,641-fold lower central nervous system exposure than what is required for efficacy against the parasite (43–47). This does not account for the half-life of the compound and the multiple dosages which are typically used during active infections. Other points to note are that most drugs are typically used in combination, and when central nervous system disease occurs, the blood-brain barrier is typically leaky, which may allow more compound to cross into the brain compared to healthy individuals. Other strategies of getting therapeutic agents into the brain can be intrathecal injections, which have been reported for drug administration for difficult-to-treat meningitis cases.

Recent studies also reported the amoebicidal activity of nitroxoline against *N. fowleri*, a causative agent of primary amoebic meningoencephalitis. Based on the observation of condensed chromatin and damaged mitochondria, nitroxoline induces apoptosis of *N. fowleri* (48). Moreover, nitroxoline poses amoebicidal effect on *Acanthamoeba* spp. in a strain-dependent manner (49), but these studies look at downstream cellular stress effects from the actual mechanism of action. Using a transcriptomic analysis of *A. castellanii* trophozoites, it was found that nitroxoline exposure increased genes functioning in oxidative stress and decreased transcripts encoding proteins functioning in mitochondrial activity (50). Collectively, nitroxoline exhibits broad-spectrum amoebicidal activity against *N. fowleri*, *Acanthamoeba* spp., and *B. mandrillaris* at varying micromolar concentrations. This indicates the urgent need to discover and develop better therapeutics against these neglected, yet almost always fatal, pathogens.

## SUPPLEMENTAL MATERIAL

Supplemental material aac.01482-25-s0001.pdf Fig. S1 to S10.

ASM does not own the copyrights to Supplemental Material that may be linked to, or accessed through, an article. The authors have granted ASM a non-exclusive, world-wide license to publish the Supplemental Material files. Please contact the corresponding author directly for reuse.
