# Supplementary material for: Drug susceptibility of a clinical isolate of Balamuthia mandrillaris, a pathogenic free-living amoeba
Source: Antimicrob Agents Chemother. 2025 Dec 10;70(1):e01482-25. doi: 10.1128/aac.01482-25 (PMC12777557; doi:10.1128/aac.01482-25)
Supplement: Supplemental material — Fig. S1 to S10. [file aac.01482-25-s0001.pdf]

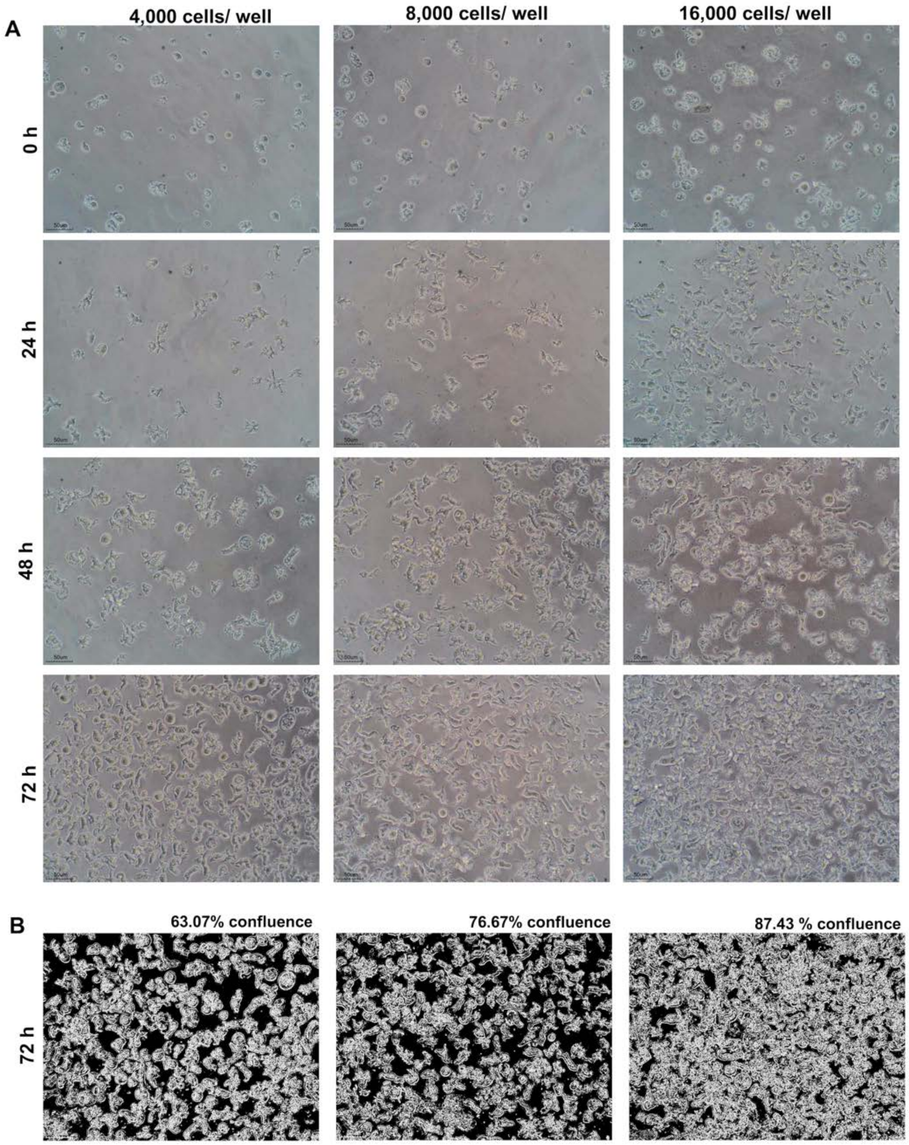

Supplementary data 1. The optimal seeding density of trophozoites. **A.** Representative images of trophozoite growth in BM-3 culture at 0 to 72 hours. The trophozoites were placed at 4,000, 8,000, and 16,000 cells in a well of the 96-well culture plate and observed under the inverted microscope every 24 hours. **B.** The percentage confluence of the trophozoites at 72 hours was measured using ImageJ. Scale bars = 50 µm.

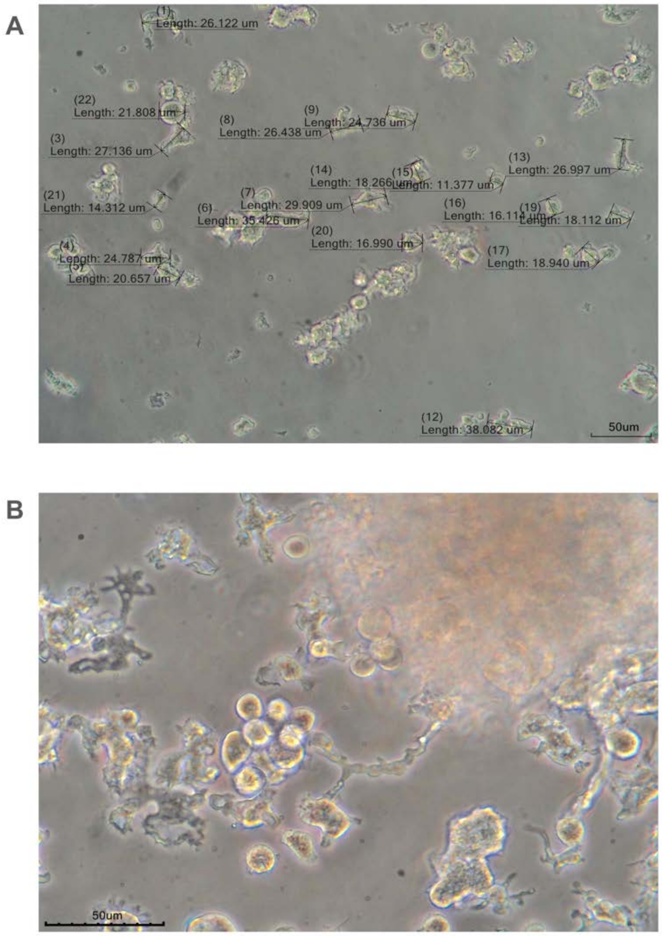

Supplementary data 2. A. Size measurement of the trophozoites. Representative image of trophozoites in BM-3 culture. ImageJ was used to measure the trophozoite length. **B.** Long-term culture of *B. mandrillaris* trophozoites in BM-3 medium. Scale bars = 50 µm.

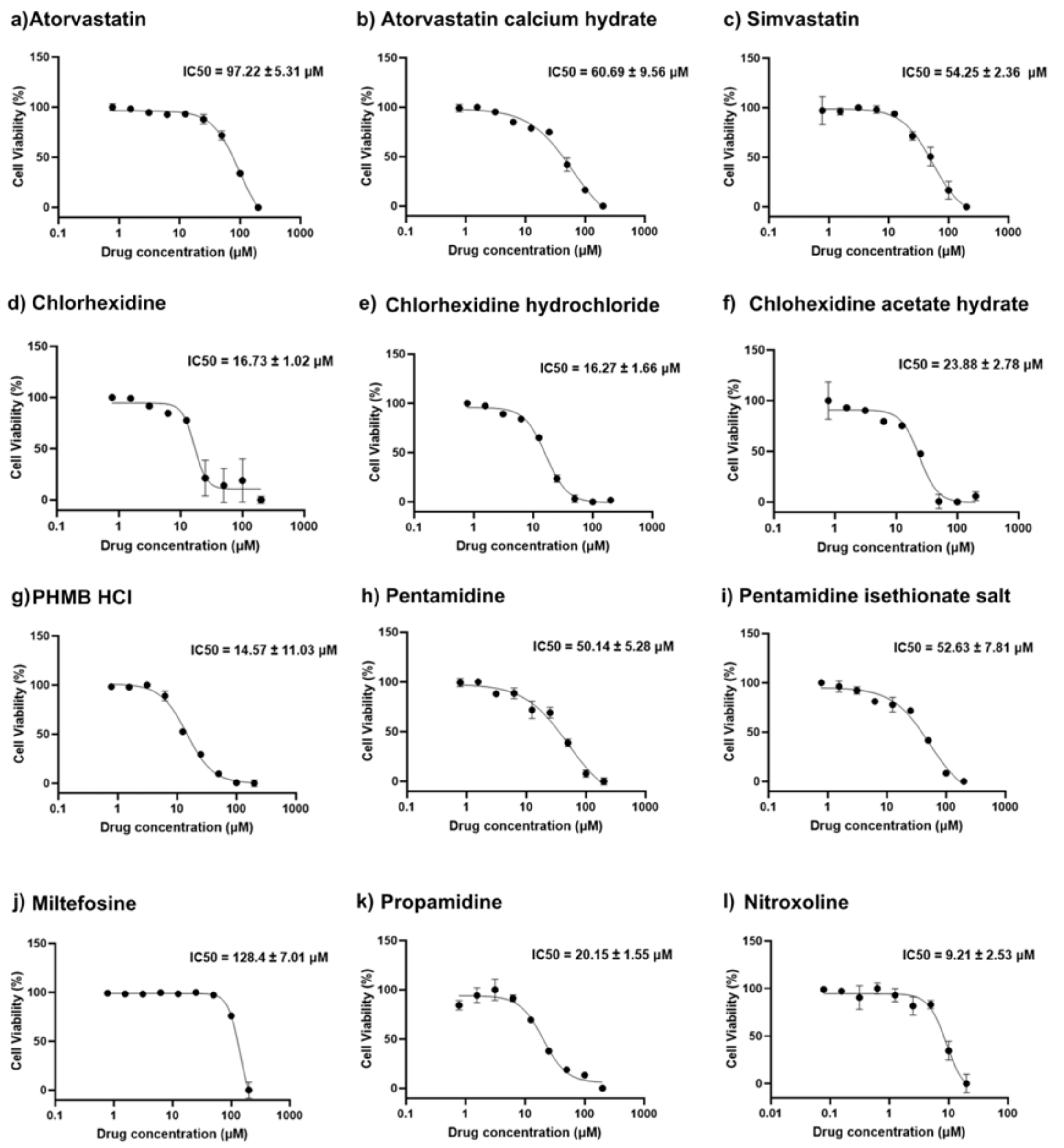

Supplementary data 3. Dose-response curves of the tested drugs. The ATP level was used as an indicator of the remaining active trophozoites. The ATP levels from the vehicle control experiments were used to calculate cell survivability. Cell survivability was calculated from the luminescence signals of the ATP by comparing them to those of the vehicle control experiment. The tested drugs include: a) atorvastatin, b) atorvastatin calcium hydrate, c) simvastatin, d) chlorhexidine, e) chlorhexidine hydrochloride, f) chlorhexidine acetate hydrate, g) polyhexamethylene biguanide hydrochloride (PHMB HCl), h) miltefosine, i) pentamidine, j) pentamidine isethionate salt, k) propamidine, and l) nitroxoline. Data for each dose were from three biological replicates and were displayed as mean±SD.

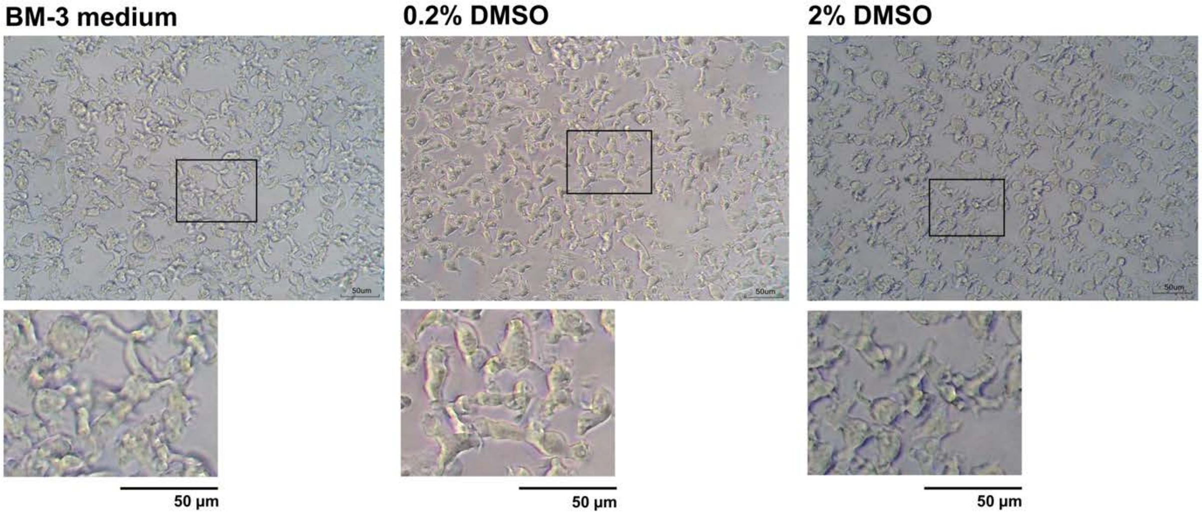

Supplementary data 4. Microscopic images of the trophozoites exposed to DMSO, the solvent for dissolving each drug, as a vehicle control experiment. Exposure to 0.2 and 2% DMSO did not alter the morphology of the trophozoites compared to the nonexposure control experiment.

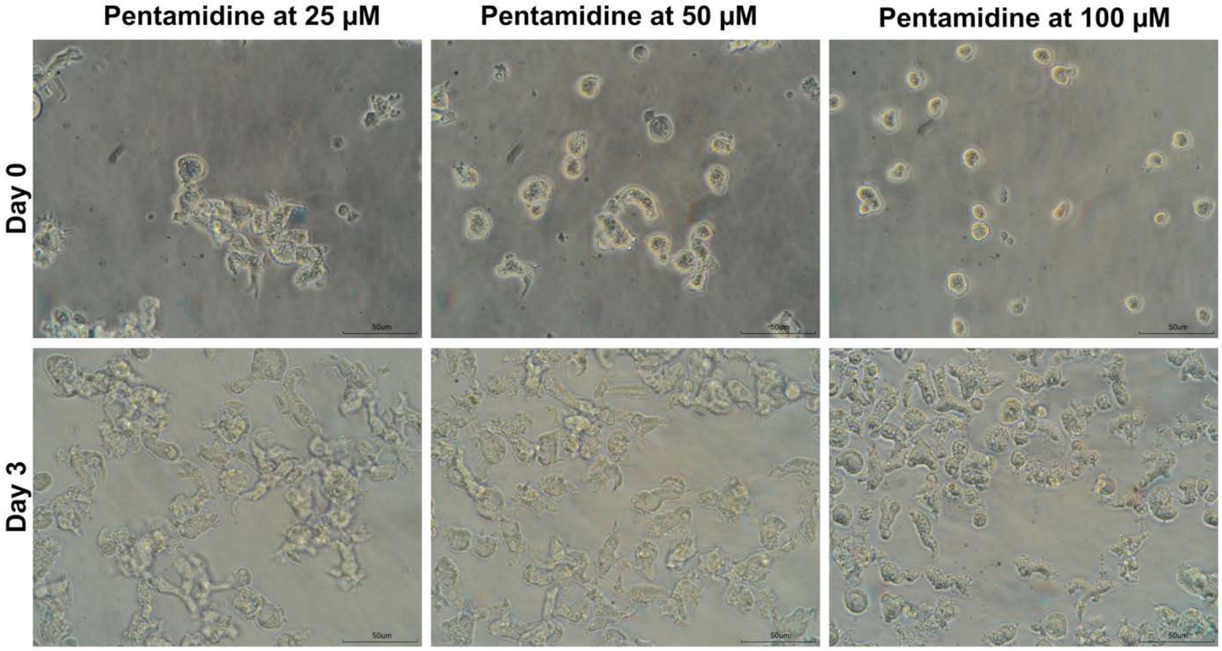

Supplementary data 5. The recrudescence of *B. mandrillaris* trophozoites after pentamidine exposure. The microscopic morphology of trophozoites was investigated from days 0 to 3 under an inverted microscope. Representative images show morphological changes, including elongation and size increase. Scale bars = 50 µm.

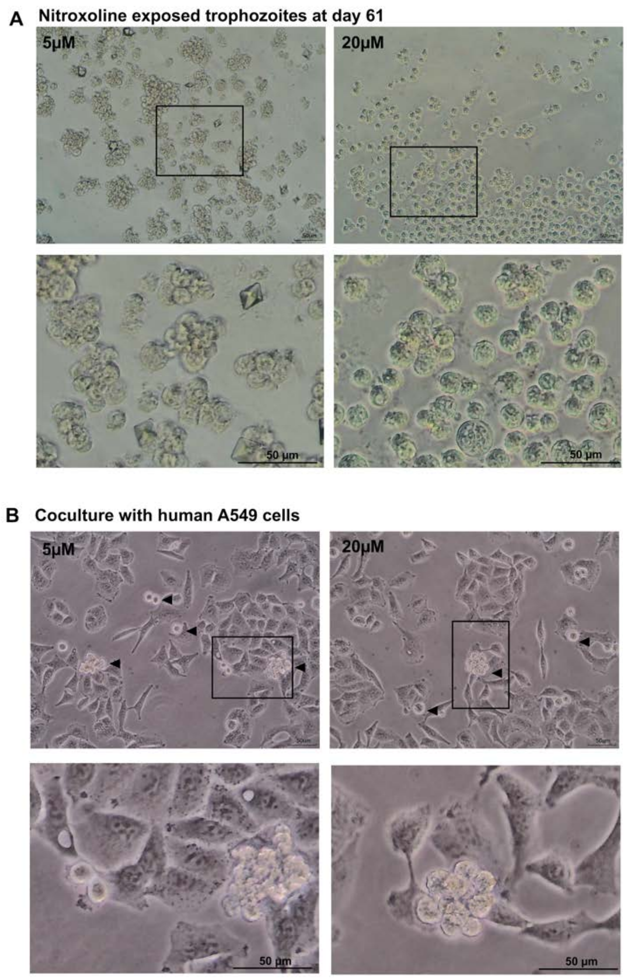

Supplementary data 6. Long-term recrudescence assay in the axenic culture. **A.** Cell morphology of nitroxoline-exposed trophozoites in the axenic BM-3 culture at day 61. After drug exposure, the trophozoites were washed to remove the drug and continuously cultured in BM-3 medium. Cell morphology was observed daily and the BM-3 medium was regularly changed up to 61 days. **B.** The coculture of the nitroxoline-exposed trophozoites with human lung carcinoma A549 cells. To confirm the loss of recrudescence, the nitroxoline-exposed trophozoites collected from the 61-day axenic culture were transferred to the coculture with human feeder cells. Scale bars = 50 μm.

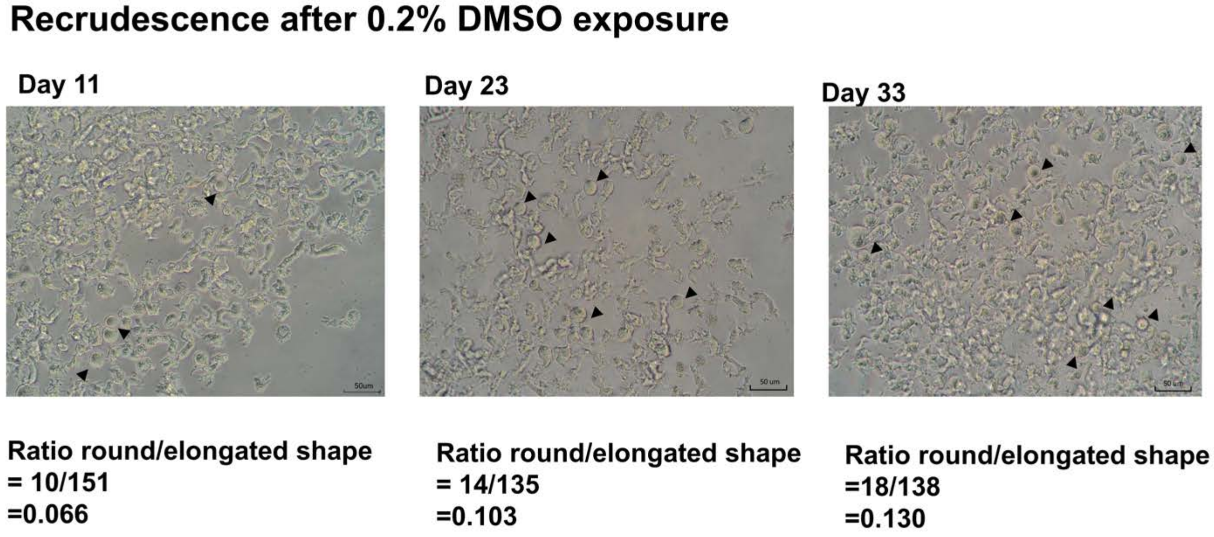

Supplementary data 7. The recrudescence of *B. mandrillaris* trophozoites after 0.2% DMSO exposure. The ratio between round and elongated shapes was estimated on days 11, 23, and 33 by counting a representative image with three readers. Scale bars = 50 µm.

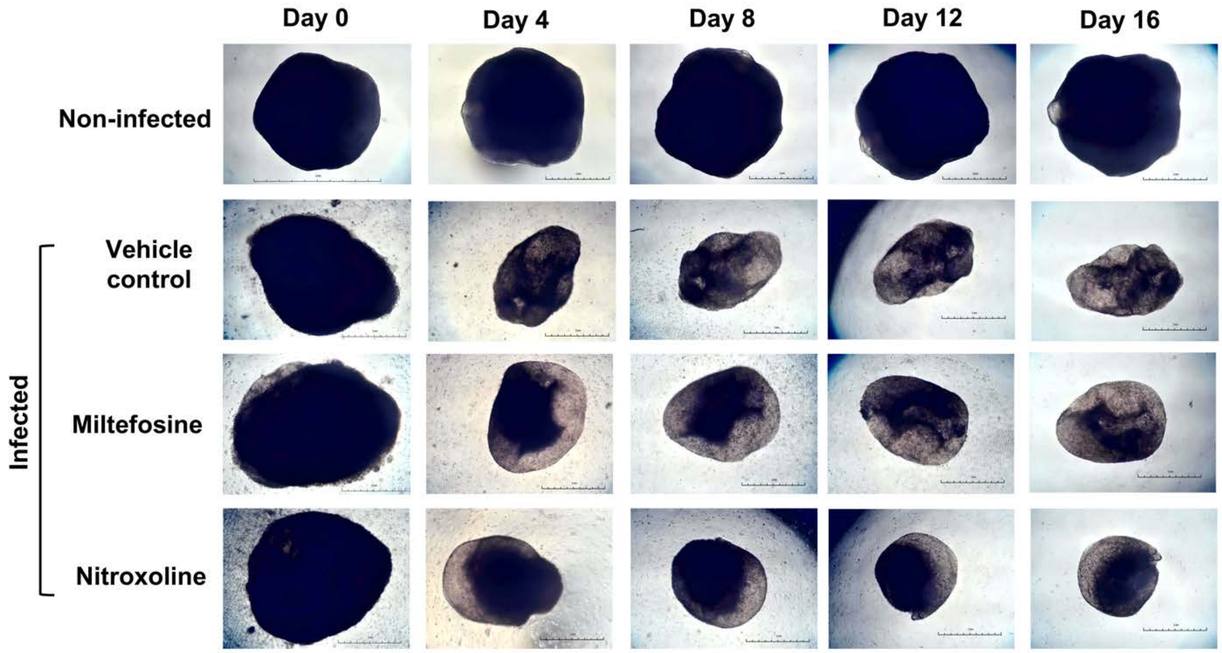

Supplementary data 8. Morphology of *B. mandrillaris*-cocultured CBs after drug exposure at different time points. Representative images of human CBs are displayed. Scale bar = 1 mm.

A

Sample	Image	Size (mm2)	Luminescent signal (RLU) No.1	No. 2	No.3	Average
Negative control (non-treated CBs)	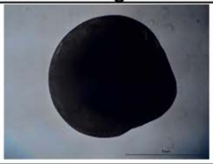	2.006	830,002	815,390	800,508	815,300
Nitroxoline	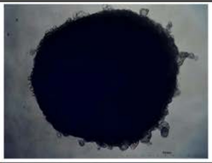	2.7	1,860,860	1,818,153	1,787,621	1,822,211
Miltefosine	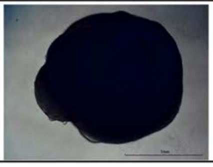	2.539	1,421,871	1,400,126	1,370,364	1,397,454

B

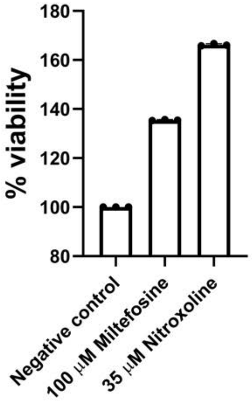

Supplementary data 9. **A.** The ATP levels of the only drug exposure in the cerebral organoid. The organoids were treated with 35 μM nitroxoline, 100 μM miltefosine, or culture media alone for 20 hours, and the ATP levels were reported as luminescent signal (RLU). Representative images of the organoids were captured under inverted microscopy. Scale bar = 1 mm. **B.** Cell survivability of drug exposure in the cerebral organoid. The luminescence signals of ATP were used to calculate cell survivability compared to that of the untreated control (media alone).

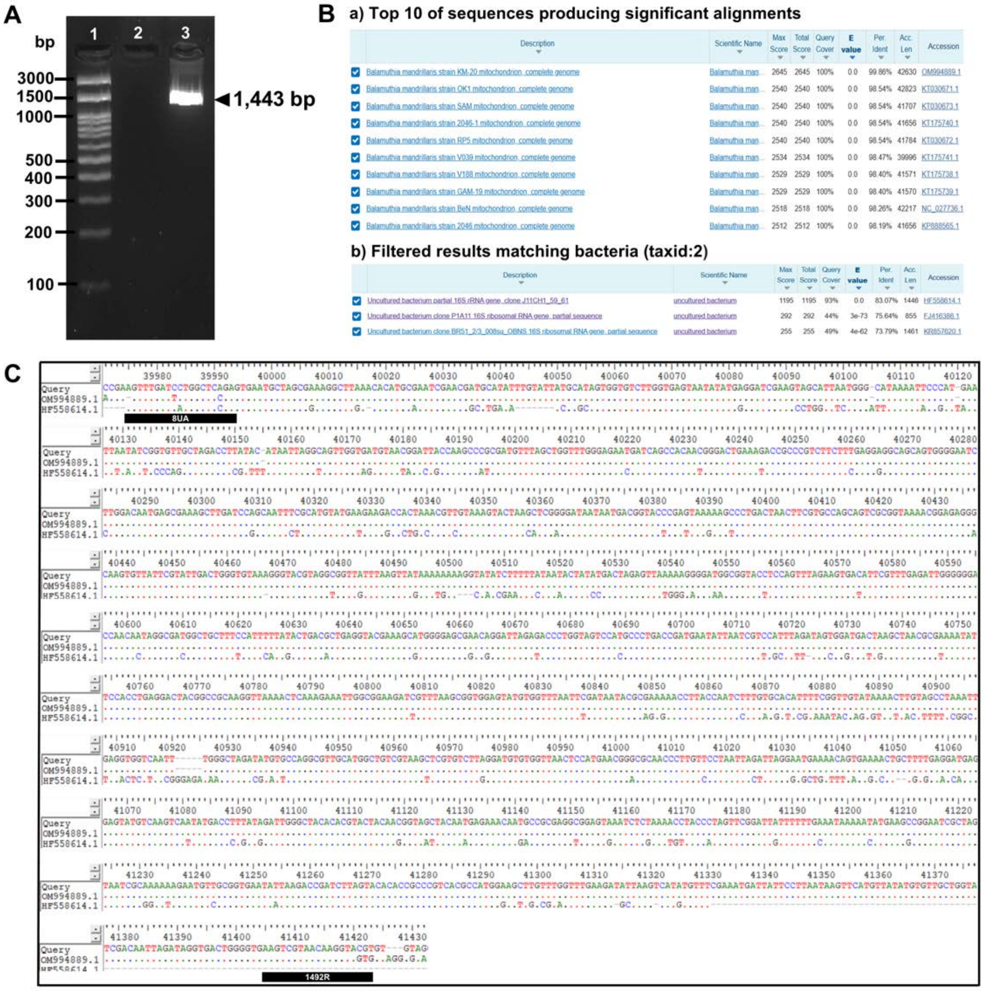

Supplementary data 10. Identification of *Balamuthia mandrillaris* with potential endosymbiotic bacteria. **A.** Agarose gel electrophoresis of the 16S rRNA PCR product. DNA amplification was performed with the 8UA forward primer (5′-AGAGTTTGATCCTGGCTCAG-3′) and the 1492R reverse primer (5′-GGTTACCTTGTTACGACTT-3′). Lane 1: 100-bp DNA ladder; Lane 2: negative control (no template); Lane 3: PCR product showing the expected ~1,443-bp amplicon. **B.** BLASTn analysis of the PCR-derived sequence. The figure shows the top 10 highest-scoring alignments to *B. mandrillaris*, each with >98.19 % sequence identity (panel a). Results were filtered to include only bacterial database matches (taxid: 2) (panel b). **C.** Multiple sequence alignment of the 16S rRNA amplicon. Sequences from *B. mandrillaris* KM-20 and uncultured bacteria, together with GenBank reference sequences (accession numbers OM994889.1 and HF558614.1), were aligned using ClustalW. Identical nucleotides are indicated by dots, and primer-binding regions for 8UA and 1492R are highlighted with black boxes.
